# Nationwide study of the impact of D2 lymphadenectomy on survival after gastric cancer surgery

**DOI:** 10.1002/bjs5.50270

**Published:** 2020-03-04

**Authors:** C.‐H. Kung, J. A. Tsai, L. Lundell, J. Johansson, M. Nilsson, M. Lindblad

**Affiliations:** ^1^ Department of Clinical Science, Intervention and Technology, Division of Surgery Karolinska Institutet Stockholm Sweden; ^2^ Department of Digestive Surgery Karolinska University Hospital Stockholm Sweden; ^3^ Department of Surgery Skellefteå County Hospital Skellefteå Sweden; ^4^ Department of Clinical Sciences Lund University Lund Sweden; ^5^ Department of Surgery Skåne University Hospital Lund Sweden; ^6^ Department of Surgery Odense University Hospital Odense Denmark

## Abstract

**Background:**

Gastrectomy including D2 lymphadenectomy is regarded as the standard curative treatment for advanced gastric cancer in Asia. This procedure has also been adopted gradually in the West, despite lack of support from RCTs. This study sought to investigate any advantage for long‐term survival following D2 lymphadenectomy in routine gastric cancer surgery in a Western nationwide population‐based cohort.

**Methods:**

All patients who had a gastrectomy for cancer in Sweden in 2006–2017 were included in the study. Prospectively determined data items were retrieved from the National Register of Oesophageal and Gastric Cancer. Extent of lymphadenectomy was categorized as D1+/D2 or the less extensive D0/D1 according to the Japanese Gastric Cancer Association classification. Overall survival was analysed and, in addition, a variety of possible confounders were introduced into the Cox proportional hazards regression model.

**Results:**

A total of 1677 patients underwent gastrectomy, of whom 471 (28·1 per cent) were classified as having a D1+/D2 and 1206 (71·9 per cent) a D0/D1 procedure. D1+/D2 lymphadenectomy was not associated with higher 30‐ or 90‐day postoperative mortality. Median overall survival for D1+/D2 lymphadenectomy was 41·5 months with a 5‐year survival rate of 43·7 per cent, compared with 38·5 months and 38·5 per cent respectively for D0/D1 (*P* = 0·116). After adjustment for confounders, in multivariable analysis survival was significantly higher after D1+/D2 than following D0/D1 lymphadenectomy (hazard ratio 0·81, 95 per cent c.i. 0·68 to 0·95; *P* = 0·012).

**Conclusion:**

This national registry study showed that long‐term survival after gastric cancer surgery was improved after gastrectomy involving D1+/D2 lymphadenectomy compared with D0/D1 dissection.

## Graphical abstract

A nationwide prospective register‐based study examined the extent of lymphadenectomy in curative gastric cancer surgery and its effects on long‐term survival.

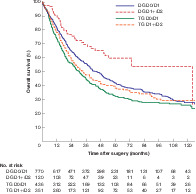

More evidence in favour of thorough lymphadenectomy

## Abstract (es)

**Antecedentes:**

En Asia, la gastrectomía con linfadenectomía D2 asociada se considera el tratamiento curativo estándar para el cáncer gástrico avanzado. Este procedimiento se ha adoptado gradualmente también en el mundo occidental a pesar de la falta de apoyo de los ensayos clínicos aleatorizados. En este estudio hemos tratado de investigar cualquier ventaja sobre la supervivencia a largo plazo tras la linfadenectomía D2 de rutina en una cohorte de base poblacional de cirugía del cáncer gástrico en un país occidental.

**Métodos:**

Se incluyeron todos los pacientes que fueron sometidos a gastrectomía por cáncer en Suecia desde 2006‐2017. Se recuperaron datos registrados prospectivamente del Registro Nacional de Cáncer de Esófago y Estómago. La extensión de la linfadenectomía se categorizó en D1+/D2 o cuando fue menos amplia en D0/D1 de acuerdo con la clasificación de la *Japanese Gastric Cancer Association*. Se analizó la supervivencia global y, además, se introdujeron diversos factores de confusión en un modelo de regresión de riesgos proporcional de Cox.

**Resultados:**

Un total de 1.677 pacientes fueron sometidos a gastrectomía, de los cuales 471 (28%) fueron clasificados como D1+/D2 y 1.206 (72%) como D0/D1. La linfadenectomía D1+/D2 no se asoció con una mayor mortalidad postoperatoria a los 30 y 90 días. La mediana de la supervivencia global para la linfadenectomía D1+/D2 fue de 41,5 meses con una tasa de supervivencia a los 5 años de 44% comparado con 38,5 meses y 39%, respectivamente, para D0/D1 (*P* = 0,116). Después de ajustar por los factores de confusión en el análisis multivariable, la supervivencia fue significativamente más alta en la linfadenectomía D1+/D2 comparada con D0/D1 (cociente de riesgos instantáneos, *hazard ratio*, HR 0,81 (i.c. del 95% 0,68‐0,95), *P* = 0,012)).

**Conclusión:**

Este estudio del registro nacional mostró que la supervivencia a largo plazo tras cirugía del cáncer gástrico mejoró después de una gastrectomía que incluya linfadenectomía D1+/D2 en comparación con la disección D0/D1.

## Introduction

Radical resection for gastric cancer remains the mainstay for cure in patients with locally advanced disease. In the West it has been shown that long‐term survival can be improved further by adding perioperative chemotherapy^1^, ^2^, ^3^, whereas in studies from Japan, South Korea, China and Taiwan, adjuvant chemotherapy is usually recommended^4^, ^5^. Classification of the extent of lymphadenectomy has changed over time, and is now classified as D0, D1 and D2 according to the Japanese Gastric Cancer Association (JGCA)^6^. Three European RCTs^7^, ^8^, ^9^ have investigated D2 lymphadenectomy compared with a less extensive D1 lymphadenectomy. No study showed a survival advantage for D2 lymphadenectomy at 5 years, but the Dutch trial^10^ was able to show a benefit at 15 years of follow‐up after exclusion of postoperative deaths. Despite these results, D1+/D2 lymphadenectomy has become widely used in the West. The present nationwide cohort study was undertaken to see whether there was a long‐term survival benefit for patients with gastric cancer undergoing D1+/D2 lymphadenectomy.

## Methods

This was a national quality register study including all patients in Sweden registered in the National Register of Oesophageal and Gastric cancer (NREV) from 2006 to 2017. The NREV, which has detailed clinical information on all patients with gastric cancer in Sweden, was cross‐matched with data from the Swedish National Patient Register, National Register of Education, Emigration Register and Death Register to obtain educational level, emigration status, time of death, and modified Charlson Co‐morbidity Index (CCI)^11^. The NREV has been validated previously and shown to be accurate for a variety of variables in more than 91 per cent of patients^12^. The Swedish National Patient Register has complete coverage of patient diagnostic codes in inpatient care from 1987, and for specialized outpatient care since 2001^13^. The form and structure of the data collection have been described previously^14^.

The study was approved by the Regional Ethics Committee (EPN Stockholm Dnr: 2016/1486‐32 and 2013/596‐31/3).

### Study population

All patients undergoing resection for adenocarcinoma of the stomach and gastro‐oesophageal junction cancer type III were included. Patients who had a previous gastric resection or who had undergone proximal gastrectomy or pylorus‐preserving gastrectomy were excluded, as were those who had a resection described as palliative. Patients were classified by the extent of lymphadenectomy as having a D0, D1, D1+ or D2 procedure according to the fourth English version of the JGCA treatment guidelines for distal and total gastrectomy^6^. An *a priori* decision grouped D1+ and D2 lymphadenectomies together as lymphadenectomy station criteria are similar and correspond to more radical resection, whereas D0 and D1 lymphadenectomy were grouped and analysed together as they represent limited lymphadenectomies.

### Statistical analysis

Data are presented as mean(s.d.) values or as counts with percentages. Statistical analyses were performed with the χ^2^ test or Fisher's exact test for categorical variables, and Student's *t* test for continuous variables. Survival was calculated by use of the Kaplan–Meier method and analysed with the log rank test. Multivariable analysis of factors affecting survival was done by Cox proportional hazards modelling, and presented with hazard ratios (HRs) and 95 per cent confidence intervals. Variables in the model included: age (as a continuous variable), sex, CCI (categorized as 0–1, 2 and 3 or above), ASA grade (categorized as I, II, III, IV, V or missing), clinical tumour stage according to TNM8 (grouped as stage I, II, III, IV and missing), surgical procedure (distal or total gastrectomy), multivisceral resection (includes any additional organ resection in addition to gastrectomy, categorized as no or yes), preoperative chemotherapy (no, yes or missing), educational level (categorized as 9 years or less, 10–12 years, more than 12 years or missing), and calendar year of surgery (grouped as 2006–2009, 2010–2013 and 2014–2017). The variables were chosen based on clinical importance and statistical significance in the model. Educational level was included as it has been shown to be a possible confounder for survival^15^. The significance level was set at *P* = 0·100 or less for a variable to be included in the model.

All statistical analyses were performed with IBM SPSS® Statistics version 25 (IBM, Armonk, New York, USA).

## Results

**Figure 1:**
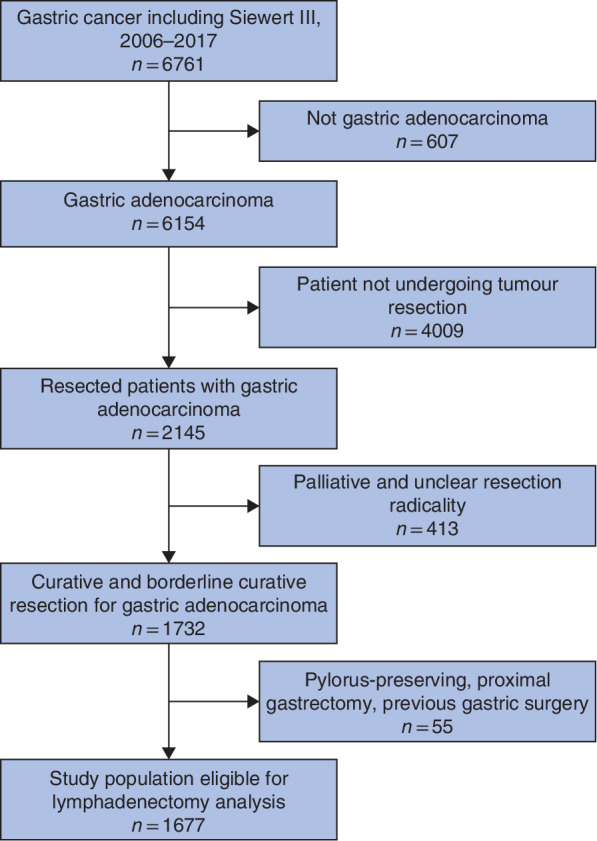
Flow chart of patient selection

A total of 6761 patients were diagnosed with gastric and gastro‐oesophageal junction type III cancer. Among these, 1677 had non‐palliative distal or total gastrectomy for adenocarcinoma (*Fig*. 1). The extent of lymphadenectomy was D0/D1 in 1206 patients (71·9 per cent) and D1+/D2 in 471 (28·1 per cent). The complete distribution was 930 D0 (55·5 per cent), 276 D1 (16·5 per cent), 126 D1+ (7·5 per cent) and 345 D2 (20·6 per cent).

Baseline characteristics of patients are presented in *Table* 1. In general, patients who had a D1+/D2 procedure were younger with a higher level of education, a slightly lower ASA grade and a more advanced tumour stage (stage III or above: 24·8 per cent compared with 14·6 per cent in the D0/D1 group). D1+/D2 lymphadenectomy became more popular during the latter part of the study period, comprising 20·8 per cent of resections in 2006–2009 *versus* 41·2 per cent in 2014–2017.

**Table 1:** Baseline characteristics of patients undergoing limited D0/D1 lymphadenectomy or more extensive D1+/D2 dissection

	D0/D1 (*n* = 1206)	D1+/D2 (*n* = 471)	Overall (*n* = 1677)	*P* †
**Age (years)** *	70(11)	65(12)	69(12)	< 0·001§
**Sex**				0·602
M	698 (57·9)	266 (56·5)	964 (57·5)	
F	508 (42·1)	205 (43·5)	713 (42·5)	
**BMI (kg/m^2^)** *	25·1(4·45)	25·2(4·43)	25·2(4·44)	0·682§
**ASA grade**				< 0·001‡
I	310 (25·7)	168 (35·7)	478 (28·5)	
II	591 (49·0)	223 (47·3)	814 (48·5)	
III	252 (20·9)	66 (14·0)	318 (19·0)	
IV	24 (2·0)	3 (0·6)	27 (1·6)	
V	0 (0)	0 (0)	0 (0)	
Missing	29 (2·4)	11 (2·3)	40 (2·4)	
**Clinical tumour stage**				< 0·001
I	402 (33·3)	120 (25·5)	522 (31·1)	
II	312 (25·9)	167 (35·5)	479 (28·6)	
III	149 (12·4)	100 (21·2)	249 (14·8)	
IV	27 (2·2)	17 (3·6)	44 (2·6)	
Missing	316 (26·2)	67 (14·2)	383 (22·8)	
**Charlson Co‐morbidity Index**				0·655
0–1	400 (33·2)	147 (31·2)	547 (32·6)	
2	195 (16·2)	83 (17·6)	278 (16·6)	
≥ 3	611 (50·7)	241 (51·2)	852 (50·8)	
**Years of education**				< 0·001
≤ 9	474 (39·3)	149 (31·6)	623 (37·1)	
10–12	455 (37·7)	193 (41·0)	648 (38·6)	
> 12	186 (15·4)	111 (23·6)	297 (17·7)	
Missing	91 (7·5)	18 (3·8)	109 (6·5)	
**Tumour location**				< 0·001
GOJ Siewert III	37 (3·1)	52 (11·0)	89 (5·3)	
Upper	39 (3·2)	34 (7·2)	73 (4·4)	
Middle	386 (32·0)	205 (43·5)	591 (35·2)	
Lower	625 (51·8)	123 (26·1)	748 (44·6)	
Whole	25 (2·1)	23 (4·9)	48 (2·9)	
Missing	94 (7·8)	34 (7·2)	128 (7·6)	
**Calendar year of surgery**				< 0·001
2006–2009	468 (38·8)	98 (20·8)	566 (33·8)	
2010–2013	469 (38·9)	179 (38·0)	648 (38·6)	
2014–2017	269 (22·3)	194 (41·2)	463 (27·6)	

Values in parentheses are percentages unless indicated otherwise;

* values are mean(s.d.). GOJ, gastro‐oesophageal junction.

† χ^2^ test, except

‡ Fisher's exact test and

§ Student's *t* test.

Surgical details are presented in *Table* 2. The distribution between total and distal gastrectomy was almost equal in the overall cohort. There was, however, a difference in the extent of lymphadenectomy related to the type of gastrectomy, with more D1+/D2 being performed in patients having a total gastrectomy (74·5 per cent) than in those having a distal gastrectomy (25·5 per cent). Preoperative chemotherapy was more commonly used in patients who subsequently underwent more extensive lymphadenectomy. Multivisceral resection included pancreatosplenectomy or splenectomy alone, as well as colectomy, adrenalectomy, cholecystectomy, and resection of the diaphragm, small bowel and liver. Multivisceral resection was associated more frequently with D1+/D2 lymphadenectomy. There was no significant difference regarding the use of laparoscopy or open surgery between the groups.

**Table 2:** Surgical details of patients undergoing limited D0/D1 lymphadenectomy or more extensive D1+/D2 dissection

	D0/D1 (*n* = 1206)	D1+/D2 (*n* = 471)	Overall (*n* = 1677)	*P* *
**Surgical procedure**				< 0·001
Distal gastrectomy	770 (63·8)	120 (25·5)	890 (53·1)	
Total gastrectomy	436 (36·2)	351 (74·5)	787 (46·9)	
**Laparoscopic surgery**				0·111
No	1171 (97·1)	450 (95·5)	1621 (96·7)	
Yes	35 (2·9)	21 (4·5)	56 (3·3)	
**Preoperative chemotherapy**				< 0·001
No	870 (72·1)	191 (40·6)	1061 (63·3)	
Yes	322 (26·7)	280 (59·4)	602 (35·9)	
Missing	14 (1·2)	0 (0)	14 (0·8)	
**Multivisceral resection**	*n* = 1197	*n* = 469	*n* = 1666	< 0·001
No	1004 (83·9)	312 (66·5)	1316 (79·0)	
Yes	193 (16·1)	157 (33·5)	350 (21·0)	
**Pancreatosplenectomy**	*n* = 1197	*n* = 469	*n* = 1666	< 0·001
No	1178 (98·4)	446 (95·1)	1624 (97·5)	
Yes	19 (1·6)	23 (4·9)	42 (2·5)	
**Splenectomy**	*n* = 1197	*n* = 469	*n* = 1666	< 0·001
No	1118 (93·4)	369 (78·7)	1487 (89·3)	
Yes	79 (6·6)	100 (21·3)	179 (10·7)	
**Emergency operation**	*n* = 1198		*n* = 1669	0·457
No	1162 (97·0)	460 (97·7)	1622 (97·2)	
Yes	36 (3·0)	11 (2·3)	47 (2·8)	

Values in parentheses are percentages.

* χ^2^ test.

D1+/D2 lymphadenectomy resulted in a significantly greater lymph node yield and, although 30‐ and 90‐day postoperative mortality was similar, there was a slightly higher overall 30‐day complication rate in the D1+/D2 group (*Table* 3).

**Table 3:** Lymph node yield and postoperative complications in patients undergoing limited D0/D1 lymphadenectomy or more extensive D1+/D2 dissection

	D0/D1 (*n* = 1206)	D1+/D2 (*n* = 471)	Overall (*n* = 1677)	*P* †
**No. of lymph nodes** *				
Distal gastrectomy	16(12) (*n* = 693)	25(15) (*n* = 106)	17(13) (*n* = 799)	< 0·001‡
Total gastrectomy	19(13) (*n* = 398)	31(18) (*n* = 301)	24(16) (*n* = 699)	< 0·001‡
**30‐day postoperative mortality**				
No	1179 (97·8)	460 (97·7)	1639 (97·7)	0·905
Yes	27 (2·2)	11 (2·3)	38 (2·3)	
**90‐day postoperative mortality**				
No	1143 (94·8)	451 (95·8)	1594 (95·1)	0·407
Yes	63 (5·2)	20 (4·2)	83 (4·9)	
**Overall complications**	*n* = 1105	*n* = 410	*n* = 1515	
No	811 (73·4)	264 (64·4)	1075 (71·0)	0·001
Yes	294 (26·6)	146 (35·6)	440 (29·0)	

Values in parentheses are percentages unless indicated otherwise;

* values are mean(s.d).

† χ^2^ test, except

‡ Student's *t* test.

**Figure 2:**
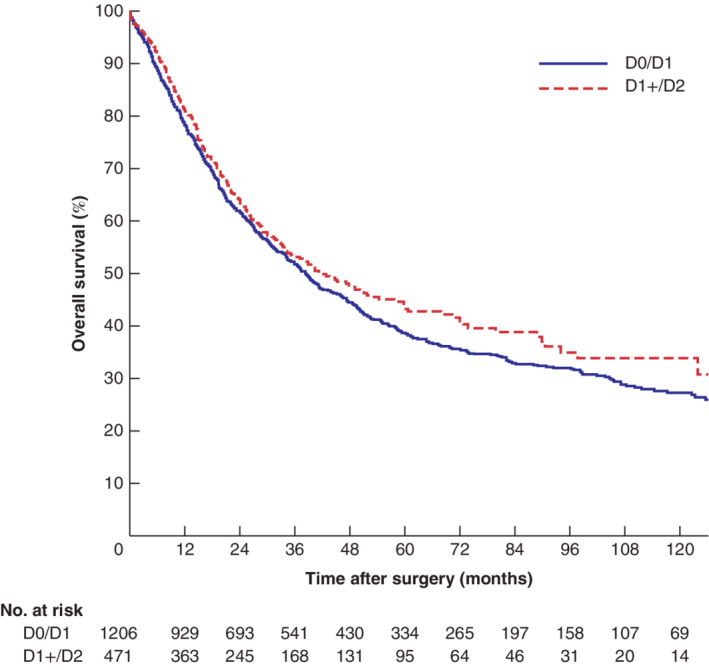
Kaplan–Meier analysis of overall survival in patients undergoing limited or more extensive lymphadenectomy D0/D1, limited lymphadenectomy; D1+/D2, more extensive dissection. *P* = 0·116 (log rank test).

Overall long‐term survival was slightly better after D1+/D2 lymphadenectomy, with a median overall survival of 41·5 months and 5‐year survival rate of 43·7 per cent compared with 38·5 months median overall survival and 5‐year survival rate of 38·5 per cent for D0/D1, although the difference was not statistically significant (*P* = 0·116) (*Fig*. 2). After adjustment for confounders, multivariable analysis indicated that survival was significantly longer after D1+/D2 lymphadenectomy compared with the D0/D1 procedure (HR 0·81, 95 per cent c.i. 0·68 to 0·95; *P* = 0·012) (*Table* 4).

**Table 4:** Cox proportional hazards analysis of the impact of lymphadenectomy on survival

	D1+/D2 *versus* D0/D1			
	Crude HR	*P*	Adjusted HR	*P*
All patients	0·89 (0·77, 1·03)	0·107	0·81 (0·68, 0·95)	0·012
Distal gastrectomy	0·61 (0·44, 0·84)	0·003	0·75 (0·54, 1·06)	0·100
Total gastrectomy	0·86 (0·72, 1·03)	0·108	0·85 (0·70, 1·04)	0·111

Values in parentheses are 95 per cent confidence intervals. Adjusted for age, sex, Charlson Co‐morbidity Index, ASA grade, clinical tumour stage, surgical procedure (in analysis of all cases), multivisceral resection, preoperative chemotherapy, educational level, and calendar year of surgery. HR, hazard ratio.

**Figure 3:**
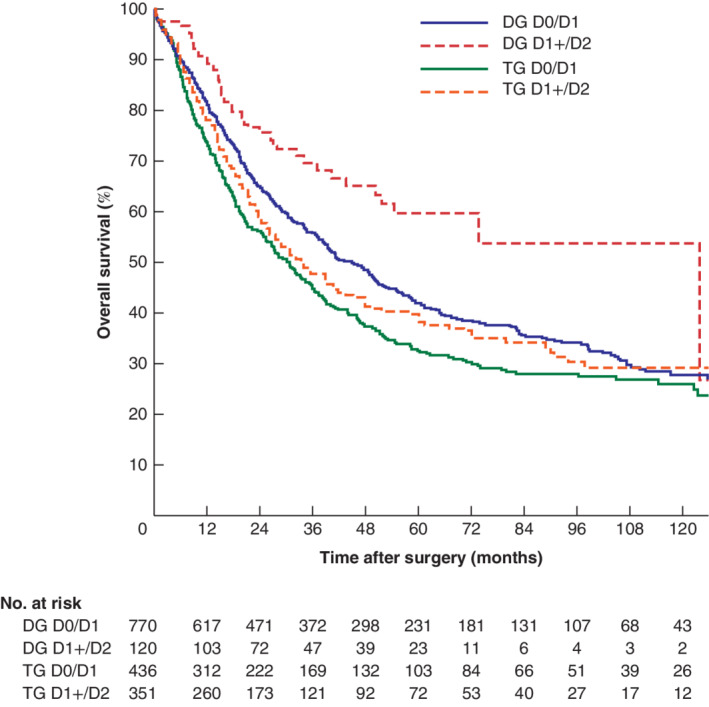
Kaplan–Meier analysis of overall survival after lymphadenectomy, stratified for surgical procedure D0/D1, limited lymphadenectomy; D1+/D2, more extensive dissection. Distal gastrectomy (DG): *P* = 0·002; total gastrectomy (TG): *P* = 0·125 (log rank test).

When stratified by surgical procedure, significantly better survival was found in patients having a distal gastrectomy and D1+/D2 lymphadenectomy compared with D0/D1 (5‐year survival rate of 59·6 *versus* 41·8 per cent respectively; *P* = 0·002). After total gastrectomy median overall survival was 33·6 months with a 5‐year survival rate of 38·7 per cent in the D1+/D2 group, compared with 30·8 months and 32·6 per cent respectively after D0/D1 lymphadenectomy (*P* = 0·125) (*Fig*. 3).

## Discussion

This study, based on a prospectively developed database from routine gastric cancer surgery in a Western population, has shown that extended lymphadenectomy (D1+/D2) performed during curatively intended gastrectomy offered better long‐term survival than limited lymphadenectomy (D0/D1). Although none of the three European randomized trials^7^, ^8^, ^9^ detected a long‐term survival advantage following gastrectomy and D2 lymphadenectomy, compared with D1 lymphadenectomy, in two early trials^16^, ^17^ D2 lymphadenectomy was associated with high postoperative morbidity and mortality, mainly due to the routine inclusion of splenectomy and pancreatic tail resection. In the latest randomized trial conducted in Italy^18^, where splenectomy and pancreatic tail resection were not standard in D2 lymphadenectomy, postoperative morbidity and mortality were substantially lower. In the Dutch trial^10^, after 15 years of follow‐up and with exclusion of postoperative deaths, a survival benefit was detected. The notion that D2 dissection can nowadays be safely carried out in Western centres was supported by a previous national registry study^14^ showing acceptable complication rates.

The traditional view is that the highest level of evidence is obtained from well designed and adequately powered RCTs. Such studies are not, however, always generalizable owing to specific patient entry criteria. On the other hand, well defined population‐based studies using data retrieved from well validated national registers, with minimal selection bias and accurate follow‐up information, reflect standard practice. The national NREV database includes detailed surgical data regarding the type of resection, including dissected lymph node stations. Most importantly, the validity and quality of the data in the NREV register have been recognized as high^12^. Some potential weaknesses must, however, be acknowledged. Detailed information on non‐surgical oncological treatments has been included only recently in the database, and was not available at the time of the present study. Although a significantly greater proportion of patients having the D1+/D2 procedure received chemotherapy, survival benefit estimates among this group remained after adjustment in the multivariable model. This suggests that chemotherapy might best be considered an adjunct to adequate lymphadenectomy as a means of improving survival after gastric cancer surgery.

Another potential problem is the risk of misclassification of the lymphadenectomy groups. There is a possibility of yielding no lymph nodes from a specific station, and of removal of nodes outside the registered extent of the resection. Information on specific lymphadenectomy stations was entered into the registry by the operating surgeon, but there has been no external validation of this. The study period covered modifications to the lymphadenectomy classifications from the second to the fourth English editions of the JGCA guidelines, with substantial change between the second and third editions^6^, ^19^, ^20^. Despite these changes, it was notable that a significantly higher lymph node harvest was found in the D1+/D2 group in the present study than in the D0/D1 group. It seems unlikely that misclassification of lymphadenectomy would have a large impact on the results of this study, as such a misclassification would most likely occur randomly, affecting each study group equally.

In Sweden, D1+/D2 lymphadenectomy was performed more commonly during total rather than distal gastrectomy, yet the greatest impact of the extent of lymphadenectomy on survival was seen in patients who had a distal gastrectomy. This difference accounts for a major part of the survival advantage in the adjusted Cox proportional hazards modelling, although it should be noted that lack of awareness of the latest JGCA guidelines on adequate lymphadenectomy during distal gastric cancer surgery may be important. Despite these limitations, this population‐based registry study of a nation's routine practice shows that long‐term survival after gastric cancer surgery is improved following gastrectomy with D1+/D2 compared with less extensive lymphadenectomy.
